# Nestin and CD133: valuable stem cell-specific markers for determining clinical outcome of glioma patients

**DOI:** 10.1186/1756-9966-27-85

**Published:** 2008-12-24

**Authors:** Mingyu Zhang, Tao Song, Liang Yang, Ruokun Chen, Lei Wu, Zhuanyi Yang, Jiasheng Fang

**Affiliations:** 1 The Neurosurgery Department of Xiangya Hospital of Central South University, Changsha, Hunan, 410078, PR China

## Abstract

**Aim:**

Gliomas represent the most frequent neoplasm of the central nervous system. Unfortunately, surgical cure of it is practically impossible and their clinical course is primarily determined by the biological behaviors of the tumor cells. The aim of this study was to investigate the correlation of the stem cell markers Nestin and CD133 expression with the grading of gliomas, and to evaluate their prognostic value.

**Methods:**

The tissue samples consisted of 56 low- (WHO grade II), 69 high- (WHO grade III, IV) grade gliomas, and 10 normal brain tissues. The expression levels of Nestin and CD133 proteins were detected using SABC immunohistochemical analysis. Then, the correlation of the two markers' expression with gliomas' grading of patients and their prognostic value were determined.

**Results:**

Immunohistochemical analysis with anti-Nestin and anti-CD133 antibodies revealed dense and spotty staining in the tumor cells and their expression levels became significantly higher as the glioma grade advanced (*p *< 0.05). There was a positive correlation between the two markers' expression in different gliomas tissues (rs = 0.89). The low expression of the two markers significantly correlated with long survival of the glioma patients (*p *< 0.05). The survival rate of the patients with Nestin+/CD133+ expression was the lowest (*p *< 0.01), and the multivariate analysis confirmed that conjoined expression of Nestin+/CD133+ and Nestin-/CD133- were independent prognostic indicators of gliomas (both *p *< 0.01, Cox proportional hazard regression model).

**Conclusion:**

These results collectively suggest that Nestin and CD133 expression may be an important feature of human gliomas. A combined detection of Nestin/CD133 co-expression may benefit us in the prediction of aggressive nature of this tumor.

## Background

Gliomas, the most frequent primary tumors in the human central nervous system (CNS), are classified into low- and high-grade glioma according to their morphological features [1]. Cells of low-grade (WHO grade I and II) gliomas are well differentiated with clear histological similarity to astrocyte or oligodendrocyte lineage. High-grade (WHO grade III and IV) gliomas are more anaplastic, with features resembling immature astrocytes, oligodendrocytes or a mixture of both types [2]. A considerable number of advances on therapeutics of malignant gliomas have been got in the last decade [3,4]. But unfortunately, surgical cure, as the basic treatment of malignant gliomas, is practically impossible to resect effectively because of their infiltrating nature and high recurrence, as this result, gliomas portend a poor prognosis [5,6]. To address this problem, many investigations have been performed and recent discoveries have shed light on the genetic events which lead to human gliomas.

Currently, a growing body of evidence suggests that gliomas may be generated from tumor stem cells (TSC) sharing many properties with those of neural stem cells (NSC) [7,8]. Nestin is an intermediate filament (IF) protein expressed in proliferating cells during the developmental stages in a variety of embryonic and fetal tissues [9]. It may be involved in the organization of the cytoskeleton, cell signaling, organogenesis, cell metabolism, and represent the proliferation, migration and multi-differentiated characteristics of multi-lineage progenitor cells [10]. CD133 (also named as Prominin) is a cell surface marker expressed on normal human NSC and acutely dissociated brain tumor cells [11]. Five alternative promoters, three of which are partially regulated by methylation, drive the transcription of several mRNA isoforms of CD133. Its localization is in membrane protrusions, which suggests an involvement in the dynamic organization of membrane protrusions and therefore in the mechanisms influencing cell polarity, migration and interaction of stem cells with neighbouring cells and/or extracellular matrix [12].

It has been reported that TSC expressing the markers Nestin and CD133 in a variety of brain tumors [13]. Analyses on their expression may be useful in predicting behavior of tumors in order to identify which tumors are most amenable to therapy. Therefore, the purpose of this study is to determine the correlation of Nestin and CD133 expression with the grading of gliomas and their predictive efficiency in clinical outcome of patients.

## Materials and methods

### Patients and Tissue Samples

Our study was approved by the Ethics Committee of Xiangya Hospital of Central South University, Changsha, Hunan, P.R.China. We searched the surgical pathology database of neurosurgery department of Xiangya Hospital of Central South University, Changsha, Hunan, P.R.China for cases from July 11^th ^1998 to July 7^th ^2001, and identified 125 consecutive, surgically resected gliomas. None of the patients had undergone chemotherapy or radiotherapy before surgery. There were 85 males and 40 females (2.125:1), with a mean age of 48.13 years (range form 8 to 75 years). The World Health Organization (WHO) defines four grades of gliomas with increasing histologic abnormality [11]. Tumors in grades II to IV are diffuse and invade normal brain. Grade II tumors are also called "low-grade gliomas," grade III tumors are called anaplastic, and grade IV tumors are known as glioblastoma multiforme (GBM). According to this standard, there were 56 low- (WHO grade II), 69 high- (WHO grade III, IV) grade gliomas. 10 normal brain tissues were used as control samples for the immunohistochemical analysis.

All patients were given a follow-up ranging from one year to seven years. The survival periods of the patients with gliomas were calculated and the date of the initial surgery was set as zero. All the patients died of other diseases but not gliomas or unexpected events were excluded from the case collection.

## Methods

### Immunohistochemical Staining

The expression levels of Nestin and CD133 in glioma tissues were analyzed by immunohistochemical staining. Tissues were fixed in 10% buffered formalin and embedded in paraffin. Commercially available monoclonal antibodies to Nestin and CD133 (Santa Cruz™, USA and Novocostra) were used. Immunohistochemical staining was carried out using the avidin-biotin method and a commercially available kit (Vectastain Elite ABC kit, Vector Laboratories, Burlingame, CA). One paraffin-embedded block of glioma tissue was selected from each case and cut into 4 μm sections. Deparaffinized sections were treated with methanol containing 3% hydrogen peroxide for 10 min before conducting antigen retrieval using a microwave oven at 95°C for 5 min and cooling at 25°C for 2 hours. After washing with PBS, blocking serum was applied for 10 min. The sections were incubated with an anti-Nestin monoclonal antibody (1:500) and anti- CD133 monoclonal antibody (1:100) overnight at 4°C. Negative control sections were incubated with PBS instead of the primary antibody. After washing in PBS, a biotin-marked secondary antibody was applied for 10 min followed by a peroxidase-marked streptavidin for an additional 10 min. The reaction was visualized by using 3, 3'-diaminobenzidine tetrahydrochloride. The nuclei were counterstained with hematoxylin. Positive and negative immunohistochemistry controls were routinely used. Reproducibility of staining was confirmed by reimmunostaining via the same method in multiple, randomly selected specimens.

Immunohistochemical staining for Nestin and CD133 was scored for the tumor cells. The number of positive-staining cells showing immunoreactivity on the cell membranes and cytoplasm in ten representative microscopic fields was counted and the percentage of positive cells was calculated. The score of Nestin and CD133 immunoreactivitiy in tissue sections was evaluated as negative (0) when no positive cells were observed within the tumor, weak (1+) when < 30% of the tumor cells were positive, moderate (2+) when 30%~60% of the tumor cells were positive, and strong (3+) when > 60% of tumor cells were positive [14,15].

### Statistical Analysis

The software of SPSS version12.0 for Windows (SPSS Inc, IL, USA) and SAS 9.1 (SAS Institute, Cary, NC) was used for statistical analysis. Continuous variables were expressed as $\bar{X}$ ± *s*. Statistical analysis were performed with Fisher's exact test for any 2×2 tables, Pearson χ^2 ^test for non- 2×2 tables, chi-square trend test for ordinal datum, Kaplan-Meier and Cox Regression analysis for the question of survival analysis. The Spearman correlation was calculated between the expression levels of Nestin and CD133 in glioma tissues. A difference between means was considered significant if the *p *value was less than 0.05.

## Results

### Expression of Nestin and CD133 in Human Glioma tissue

**Figure 1:**
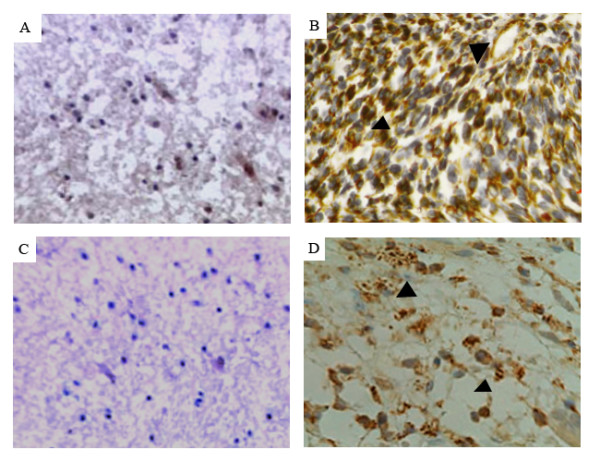
**Immunohistochemical analysis for anti-human Nestin and anti-human CD133 antibodies**. Paraffin-embedded sections of representative gliomas and normal brain tissues were stained with the antibodies against human Nestin and CD133. The photographs of A and C are normal brain tissues which showed negative or weak staining for Nestin and CD133, respectively. In contrast, the glioblastomas that have high expression levels of Nestin and CD133 with dense and spotty staining were shown in B and D, respectively.

The expression and location of Nestin and CD133 in the 125 patients of primary gliomas were examined using immunostaining analysis. The positive expression rates of Nestin (103/125, 82.4%) and CD133 (98/125, 78.4%) in patients with gliomas were higher than those in normal brain tissues (both 1/10, 10%) significantly (p < 0.001, Table 1). The two markers expression occurred mainly on the cell membrane and in the cytoplasm, which is similar to the results from previous studies [16]. Representative pictures of immunohistochemistry staining of Nestin and CD133 are shown in Figure 1.

**Table 1:** Expression of Nestin and CD133 in human gliomas and normal brain tissues

Groups	Cases (n)	0 (n, %)	1+~2+ (%)	3+ (%)	P
Nestin					
Gliomas	125	22 (17.6)	68 (54.4)	35 (28.0)	< 0.001
Normal	10	9 (90.0)	1(10%)	0 (0)	
CD133					
Gliomas	125	27 (21.6)	64 (51.2)	34 (27.2)	< 0.001
Normal	10	9 (90.0)	1(10%)	0 (0)	

### Correlation of Nestin and CD133 expression with the clinical grading of human gliomas

The expression levels of Nestin and CD133 in human gliomas tissues with different clinical grading were shown in Table 2. To evaluate whether a correlation between the expression of Nestin, CD133 and the pathological grades of gliomas could be observed a bi-variate correlation analysis (Pearson correlation coefficients) was done. Statistically significant correlations between the percentages of immunostained cells and pathological grades of gliomas were found for Nestin (c_p _= 0.592, *p *< 0.01) and CD133 (c_p _= 0.563, *p *= 0.01), which means that with higher malignant grades of gliomas, higher protein expression could be found. Furthermore, the Spearman correlations (r_s_) were 0.89 (*p *= 0.02) indicating that the expression level of Nestin was positively correlated with that of CD133 significantly.

**Table 2:** Nestin and CD133 expression in human gliomas tissues with different clinical grading

Clinical Grading	NO.	Nestin (n, %)			CD133 (n, %)		
		
		0	1+~2+	3	0	1+~2+	3
*Low-grade tumors*	*56*	*16 (28.6)*	*32 (57.1)*	8(14.3)	*20 (37.1)*	*27 (48.2)*	9(16.1)
Astrocytoma	18	7 (38.8)	11 (61.1)	0 (0)	9 (50.0)	9 (50.0)	0 (0)
Ependymoma	15	6 (40.0)	8 (53.3)	1 (6.7)	7 (46.7)	7 (46.7)	1 (6.7)
Oligodendroglioma	11	2 (18.2)	8 (72.7)	1 (9.1)	3 (27.3)	7 (63.6)	1 (9.1)
Oligodendroastrocytoma	4	1 (25.0)	1 (25.0)	2 (50.0)	1 (25.0)	1 (25.0)	2 (50.0)
Pilocytic astrocytoma	8	0 (0)	4 (50.0)	4 (50.0)	0 (0)	3 (37.5)	5 (62.5)
*High-grade tumors*	*69*	*6(8.7)*	*36 (52.2)*	*27 (39.1)*	7(10.1)	*37 (53.6)*	*25 (36.2)*
GBM	48	4 (8.3)	28 (58.3)	16 (33.3)	5 (10.4)	29 (60.4)	14 (29.2)
Anaplastic astrocytoma	11	2 (18.2)	6 (54.5)	3 (27.3)	2 (18.2)	7 (63.6)	2 (18.2)
Malignant oligodendroglioma	6	0 (0)	1 (16.7)	5 (83.3)	0 (0)	1 (16.7)	5 (83.3)
Malignant ependymoma	4	0 (0)	1 (25.0)	3 (75.0)	0 (0)	0 (0)	4 (100.0)

### Prognostic implications of Nestin and CD133 expression in human gliomas

**Figure 2:**
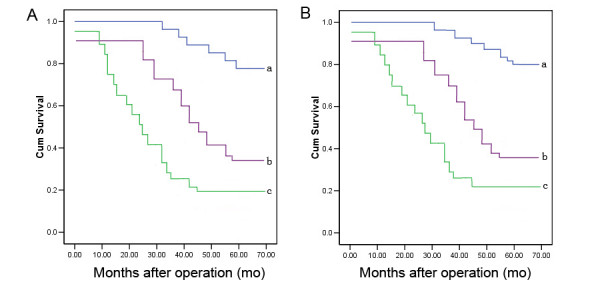
**Kaplan-Meier survival curves for Nestin (A) and CD133 (B) expression in gliomas tissues**. 'a', categorized by negative Nestin or CD133 expression; 'b', categorized by weak~moderate positive Nestin or CD133 expression; 'c', categorized by strong positive Nestin or CD133 expression. Survival was significantly poor for patients with strong positive Nestin or CD133 expression than those with negative expression (both *p *< 0.01).

The association of Nestin and CD133 expression with the 5-year survival rate of patients with gliomas was analyzed using Kaplan-Meier analysis. The patients with positive Nestin and CD133 were both categorized into three groups: 0, 1+~2+ and 3+. The Chi-square value by Mantel-Cox indicated a significant difference among different groups with regard to the expression status of Nestin and CD133 (*p *< 0.01) (Table 3, Figure 2A and 2B). The results by pairwise comparisons showed that there is a significant difference in survival rates between patients with Nestin and CD133 strong positive expression and any of the other two groups (*p *< 0.01).

**Table 3:** Prognostic value of Nestin and CD133 expression by Kaplan-Meier analysis

		5-year survival rate		
			
TYPE	Total N	n	Percent (%)	*p*
Nestin				

0	22	19	86.4	< 0.01
1+~2+	68	36	52.9	
3+	35	7	20.0	
CD133				
0	27	22	81.5	< 0.01
1+~2+	64	31	48.4	
3+	34	7	20.6	

Furthermore, according to the conjoined expression of Nestin/CD133, the patients were categorized into four groups: Nestin-/CD133-, Nestin-/CD133+, Nestin+/CD133-, and Nestin+/CD133+. Among the four groups Nestin+/CD133+ patients had the poorest prognosis. Using Cox regression analysis of the 125 patients, conjoined expression of Nestin+/CD133+ and Nestin-/CD133-, clinical grading seemed to be independent prognostic indicators. (*p *< 0.01, *p *< 0.01 and *p *= 0.02, respectively, Table 4).

**Table 4:** Prognostic value of Nestin/CD133 conjoined expression in multivariate analysis by Cox Regression

					95.0% CI for Exp(B)	
					
	Wald	df	*p*	Exp(B)	Lower	Upper
Nestin-/CD133-	23.06	3	<0.01			
Nestin-/CD133+	1.08	1	0.18	2.33	0.58	2.82
Nestin+/CD133-	0.92	1	0.21	0.89	0.33	1.54
Nestin+/CD133+	24.32	1	< 0.01	5.06	2.17	9.85
Clinical grading	16.18	1	0.02	1.98	1.23	3.89

## Discussion

To our knowledge, malignant gliomas are highly recurrent tumors even after surgery, chemotherapy, radiation and immunotherapy. Ionizing radiation represents the most effective therapy for gliomas but radiotherapy remains only palliative because of radioresistance. In the last decades, the treatment strategies for gliomas have not changed appreciably because of the limited understanding of the biology of the disease. Several recent reports suggest that normal and TSCs share the expression of several markers, the ability for self-renewal and differentiation, and signalling pathways involved in the regulation of cellular survival, proliferation [17,18]. It also has been demonstrated that TSCs exist in high-grade brain tumors and could be isolated from them. Nevertheless, little is known about the expression of these markers in solid brain tumors, especially in relation with the malignant grades of these tumors. To address this problem, we investigate, in this study, the expression of two TSC markers – Nestin and CD133, which are the most accredited markers for the identification of NSCs and have been used to fundamentally reveal the biological properties of TSCs, on protein level. In our small series of cases, Nestin and CD133 expression was associated with a poor prognosis and correlated better with clinical course than the histological grading. While the prognostic significance of the histological diagnosis strongly depends on the experience of the respective neuropathologist, analysis of both available data sets revealed that Nestin and CD133 expression was superior in predicting the patient's survival.

Nestin belongs to class VI of IFs, produced in stem cells in the mammalian CNS during development. It is a marker of proliferating and migrating cells [19]. IFs as the cytoskeleton constituents are involved in the control of cell morphology, adhesion and proliferation. When differentiation starts, cells that exit the cell cycle down-regulate Nestin and subsequently up-regulate alternative IFs such as neurofilaments in committed neurons, and GFAP in glial precursors [20]. Down-regulated Nestin may be re-expressed in the adult organism under certain pathological conditions such as brain injury, ischemia, inflammation and neoplastic transformation [21]. Nestin has been detected in brain tumors such as pilocytic astrocytomas and malignant gliomas including GBM [22-25]. IF is linked to enhanced motility and invasion in a number of different cancer subtypes. The expression of Nestin in different astrocytoma cell lines has been related to a migratory cell phenotype with increased motility and invasiveness of different astrocytoma cell lines [23]. Moreover, Dahlstrand, et al. showed high Nestin expression in high malignant tumors such as GBM when compared to less anaplastic glial tumours, which assigns to Nestin a role as new potential prognostic marker for glioblastomas [26]. In addition, Nestin has been also identified in the cell nucleus of tumor cell lines obtained from glioblastoma patients [27]. In our work, in agreement with literature data, Nestin was expressed more frequently in higher malignant grade gliomas by tumor cells, being predictive of a significantly lower 5-year survival rate.

CD133/prominin is originally found on neuroepithelial stem cells in mice. It has been isolated from hematopoietic stem cells by an antibody recognizing AC133 [28]. In general, CD133 is present in different types of stem cells and several cancers, and is down-regulated in differentiated cells [29]. CD133 localization in membrane protrusions suggests an involvement in the dynamic organization of membrane protrusions and therefore in the mechanisms influencing cell polarity, migration and interaction of stem cells with neighbour cells and/or extracellular matrix, but experimental data are currently lacking [30]. In addition, it is not known whether CD133 has a role in self-renewal and differentiation of stem cells, which has important implication in cancerogenesis. Our study investigated CD133 expression, yielding results similar to those reported by Dagmar [11] for CD133 (namely, a predictive value for a worse outcome in high-grade oligodendroglial tumor patients displaying positivity for CD133 expression).

In conclusion, Nestin and CD133 expression may be a potential indicator of the biological aggressiveness of gliomas. With its disease specificity and response to treatment demonstrated in further analysis, Nestin and CD133 can be considered as markers of tumor burden and recurrence in human gliomas.

## Abbreviations

CNS: Central nervous system; NSC: Neural stem cell; TSC: Tumor stem cell; NBT: Normal brain tissue; GFAP: Glial fibrillary acidic protein; WHO: World Health Organization; IF: Intermediate filament; PBS: Phosphate-buffered saline

## Competing interests

The authors declare that they have no competing interests.

## Authors' contributions

MZ and TS carried out the experiment of this manuscript and drafted the manuscript; LY, RC, and LW participated the experiment and revised the manuscript, ZY and JF participated in the design of the study and and approved the final manuscript.
